# Efficacy and Safety of "Coronary Artery Bypass Graft Angiography" with Right Transradial Access *versus* Left Transradial Access and Femoral Access: a Retrospective Comparative Study

**DOI:** 10.21470/1678-9741-2018-0270

**Published:** 2019

**Authors:** Yakup Balaban, Mustafa Haluk Akbaş, Merih Leventyüz Akbaş, Ali Özerdem

**Affiliations:** 1 Department of Cardiology, Vm Medicalpark Kocaeli Hospital, Başiskele, Kocaeli, Turkey.; 2 Department of Cardiology, Cihan Hastanesi, Izmit, Kocaeli, Turkey.

**Keywords:** Coronary Artery Bypass, Coronary Angiography, Mammary Arteries, Radial Artery

## Abstract

**Objective:**

Over the past 10 years, the rate of patients who have undergone coronary artery bypass graft (CABG) surgery has increased twofold in cases of coronary angiography. Today, transradial access is the first choice for coronary angiography. We aimed to compare the efficacy and reliability of radial *versus* femoral access for coronary angiography in post-CABG surgery in this study.

**Methods:**

Data from 442 patients who underwent post-CABG surgery between 2012-2017 were retrospectively compared. The right radial route was used in 120 cases, the left radial route in 148, and femoral route in 174. These three pathways were compared in terms of procedure time and fluoroscopy time, efficacy, and complication development. Comparisons among the three groups were performed with Bonferroni test for continuous variables and chi-square or Fisher's exact test for nominal variables as a binary.

**Results:**

Comparison results indicate that femoral access was better than left radial access and the left radial access was better than right radial access in terms of fluoroscopy time (10.71±1.65, 10.94±1.25, 16.12±5.28 min, *P*<0.001) and total procedure time (17.28±1.68, 17.68±2.34, 23.04±5.84 min, *P*<0.001). The left radial pathway was the most effective way of viewing left internal mammary artery (LIMA). No statistically significant differences were found among the three groups in other graft visualizations, all minor complications, total procedure and fluoroscopy time "*Except LIMA imaging*". Mortality due to processing was not observed in all three groups.

**Conclusion:**

The left radial route is preferred over right radial access for post-CABG angiography because the left radial pathway is close to the LIMA and is similar to the femoral pathway. In LIMA graft imaging, right radial access is a reliable route, even though it is not as effective as other pathways. We hope that the right radial pathway will improve with physician experience and innovations.

**Table:** 

Abbreviations, acronyms & symbols	
Ao-LAD	= Aorta-left anterior descending artery
Ao-OM	= Aorta-obtuse marginal artery
Ao-RCA	= Aorta-right coronary artery
CABG	= Coronary artery bypass graft
IM	= Intermediary artery
LIMA	= Left internal mammary artery
LSD	= Least significant difference

## INTRODUCTION

Transradial coronary angiography has recently been widely used in place of post-coronary artery bypass graft (CABG) imaging since 2010 in Europe and the larger cities of Turkey. Early recognition of coronary artery disease by noninvasive methods has led to an increase in the number people having CABG surgery in the society, and now, one in every 10 angiographies is post-CAGB angiography^[1,2]^.

In studies related to coronary angiography and percutaneous intervention that were conducted in recent years, transradial access has left its disadvantage in terms of time and performance relative to the transfemoral route, and advantageous in terms of complications^[3-5]^. Recently, radial access has been used for the purpose of carotid and renal artery angiography^[6-8]^, transradial access has been used for acute ischemic stroke intervention^[9]^, but femoral access is still advantageous from the point of view of the radiation exposure dose of the operator^[10]^. The first preference of cardiologists who have used the radial route for the first time is left transradial access for performing coronary angiography, due to the resemblance of the left radial tract to the femoral tract. Initial experience suggests that the left radial access is more advantageous than the right^[3,11]^.

Radial access has become the first choice even in the intervention of acute myocardial infarction. Recent comparative studies demonstrate that transradial access is as effective and reliable as transfemoral. It is even better than transfemoral access in terms of complications and comfort. Instead of the femoral path, the radial pathway is used, although not very common in post-CABG angiography. However, CABG imaging with the right radial pathway is not routinely performed^[12,13]^.

We aimed to investigate whether the right radial access is safe and effective by comparing the left radial route and the right radial route *versus* the femoral route in the performance of post-CABG angiography. In the situations where the left radial and femoral pathways are not appropriate, we also wanted to show that the right radial pathway can be used for post coronary bypass surgery. We anticipate no significant difference among the right, left, and femoral pathways of aortocoronary saphenous vein graft angiography. The situation for left internal mammary artery (LIMA) is different. We believe that LIMA imaging with the left radial pathway should be easier than with the femoral path because LIMA is close to it. Femoral access is the reference point added to the comparison because it is the conventional pathway. In this study, the femoral path was compared with the left radial pathway, then the left radial route was compared with the right radial route. This study intends to show that the right radial path may be an alternative to CABG angiography^[4,14,15]^.

## METHODS

### Patient Selection

From November 2012 to July 2017, 758 post-CABG surgery patients with coronary angiography presented themselves at our facility with typical chest pain, newly developed wall motion defect, acute coronary syndrome, or coronary artery disease. Patients with severe skeletal anomalies, limb loss, advanced heart failure, opaque allergy, and communication impairment, aortic diameters of 5.5 cm and above, severe aortic plaque burden, five or more grafts, or process time exceeding the confidence interval due to various reasons (vascular origin anomalies and severe vascular tortuosity and so on) were excluded from the study and not included in the comparison. 442 post-CABG surgery patients - 119 females and 323 males - and undergoing angiography, who were 44 to 96 years old, were selected for this study.

The study was conducted according to Declaration of Helsinki, considering ethical principles for medical research involving human subjects. Patients were asked to sign an informed consent form since it can be used in a retrospective study. In this way, all patients participating in the study gave written, informed consent and authorized local ethics committee in clinical research to approve the study.

In our clinic, the preferred way is the left radial pathway for post-CABG patient's angiography; the second choice is femoral access, when the left radial pathway is not appropriate (due to radial artery occlusion, obesity, or in patients where the left radial artery is used as a graft). The next option is the right radial pathway; it was used in patients who did not prefer for the femoral pathway or where the femoral pathway was not appropriate (patients who underwent femoral artery operation, femoral artery graft, undergoing femoral artery endovascular intervention, and the orthopneic patients).

### Study Protocol

Patient demographic data, epicrisis information, and resume information were transferred to the statistical program (SPSS v11, Inc., Chicago, IL, USA) and reviewed individually. The femoral route was used in 174 of these patients, while the left radial route was used in 148 and the right radial route was used in 120.

### Outcome Measure

The primary endpoint of this study was the total procedure time started after the sheath was inserted and terminated after pulling the catheter. Fluoroscopy time was assessed because it correlated with the cumulative radiation dose. Amount of opaque material used correlates to the amount used for imaging purposes in each process for each case. Graft imaging success correlates to, the determined number of grafts in each group attached to the native vessels. Finally, angiography was performed by using opaque material from the first point of grafts in the aorta. The ratio of the number of selective viewing in each group to the number of grafts was considered as the basis for comparison. We did not know in which patient the LIMA was used as a graft, so we displayed LIMA in each patient. LIMA efficacy and LIMA imaging success were recorded for each case, and complication development - local hematomas, severe hematoma, hemorrhage, radial artery spasm, radial artery occlusion, pseudoaneurysm, cerebrovascular accident, mortality and morbidity - were recorded during the first month after the procedure.

### Angiography Protocol

**Fig. 1:**
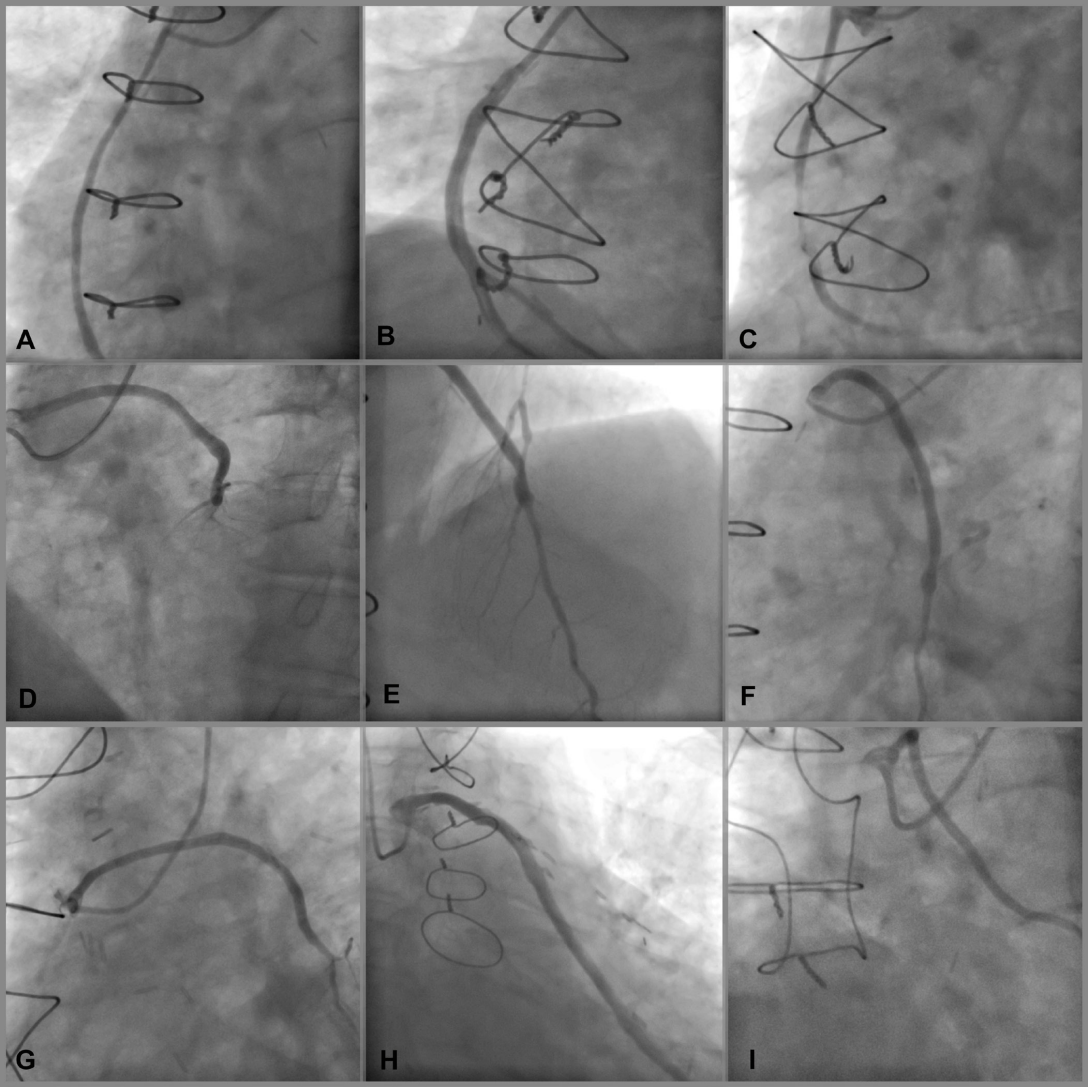
The aorta coronary bypass graft angiography with right transradial access. A, B, C = aorta-right coronary artery graft angiography. D, E, F, G = aorta-left anterior descending artery (LAD) and diagonal artery graft angiography. H, I = aorta-obtuse marginal artery graft angiography.

**Fig. 2:**
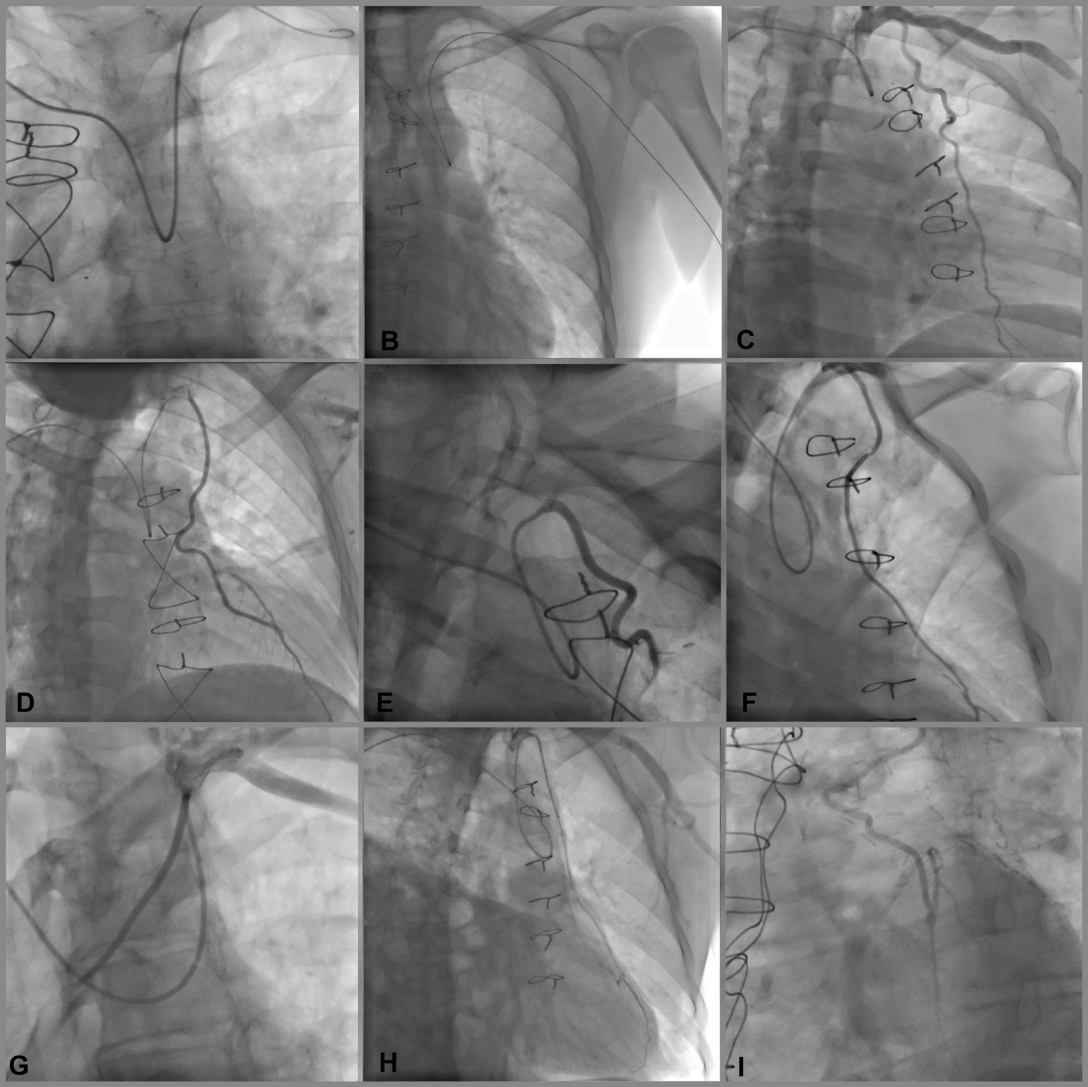
The left internal mammary artery (LIMA) angiography with right transradial access. A, B: The 0.35 guidewire and catheter moving forward from right radial artery to left subclavian artery for LIMA imaging. C, D, E, F, G, H, I; LIMA angiography images with right transradial access.

Patients with palpable right and left radial arteries were subjected to the Allen's test and Barbeau's test. In patients who underwent Barbeau's test, and whose right and left radial angiographies were not appropriate, the procedure was performed on the femoral route. In patients who were eligible for radial access, the radial region was anesthetized with 5% prilocaine, 1 mg nitroglycerin solution. 5F sheet was preferred for radial angiography because it was more comfortable with less radial arterial occlusion and spasms than 6F^[16]^. The radial artery was cannulated with 5F Terumo(r) glidesheath(tm). Then, left and right coronaries were displayed with diagnostic catheters from Boston(r) and Alvimedica(r). "Amplatz Left 1" and "Amplatz Left 2" were used if the Judkins catheters were not sufficient to display the aortocoronary bypass grafts, except for LIMA (Figures 1 and 2).

Medtronic(r)'s IMA catheter was used for LIMA imaging on the left radial and femoral route.

**Fig. 3:**
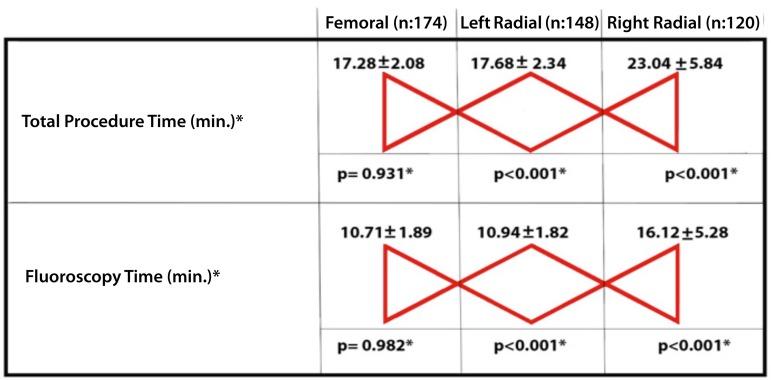
Post-hoc analysis of significantly different variables among three groups. *They were analyzed by one way ANOVA Test (95% confidence interval) with Bonferroni method (alpha 0.05).

To view the LIMA with the right radial pathway at the beginning of the procedure, firstly, aortography was performed to determine the type of aorta. LIMA imaging with right radial access is often possible in all aortic types except for type IV, where the incidence is between 3-6%, including "arteria lusoria". After the course of aortography, we engaged the left subclavian artery with the 3.5 JL catheter, then pushed the 0.35 guidewire towards the left brachial artery followed by JL catheter exchange with IMA catheter. LIMA can then be displayed with several rotation movements. In anomalous cases, if we cannot engage grafts with existing catheters, we were able to achieve imaging by catheter reshaping in the laboratory environment^[17-19]^ (Figure 3, Videos 1 and 2).

**Video 1:**
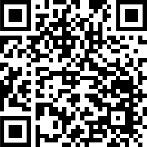

**Video 2:**
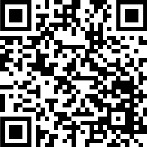

After sterilization of the femoral site for femoral angiography, local anesthesia with 5% prilocaine was applied to the femoral region. The femoral artery was cannulated with a 6F sheet. The right and left coronaries were displayed, followed by aortocoronary bypass grafts visualized with the right diagnostic catheter. LIMA was displayed with IMA catheter (Figure 1).

### Statistical Method

Continuous variables are presented as means ± standard deviations and 95% confidence intervals were determined. Nominal variables were presented as numbers and percentages. Comparisons between the approaches were performed with Student's *t*-test and one-way ANOVA for continuous variables and Chi-squared or Fisher's exact test for nominal variables. A 2-sided *P*-value less than 0.05 was considered significant. The groups with significant differences compared to continuous variables were *post hoc* analyzed. The *post hoc* analysis was performed using one-way ANOVA test with least significant difference (LSD) and Bonferroni methods'. Statistical Package for the Social Sciences version 11.0 (SPSS, Inc., Chicago, IL, USA) was used to analyze data.

## RESULTS

The demographic data, procedural data, procedure time, scopy time, selective imaging success, amount of opaque material used and complication numbers have been compared among the 174 patients that underwent post-CABG surgery angiography with the femoral route, the 148 patients with the left transradial route and the 120 patients with the right transradial route.

A total of 442 CABG patients undergoing angiography were included in the study. Female/male ratio was similar for the three groups in the femoral, left radial, and right radial groups, respectively: 45 (25.9%), 42 (28.4%), 22 (18.3%), *P*=0.149. Groups were similar in terms of average age (67.03±7.95, 68,55±9.72, 67.13±9.49 years old, *P*=0.263); number and proportion of hypertensive patients [156 (89.7%), 132 (89.2%), 109 (90.8%), *P*=0.903]; number of diabetic patients [92 (52.9%), 75 (50.7%), 62 (51.7%), *P*=0.925]; hyperlipidemic patient count [148 (81.1%), 123 (83.1%), 91 (75.8%), *P*=0.117]; renal failure [4 (2.3%), 4 (2.7%), 7 (5.8%), *P*=0.110]; smokers count [16 (9.2%), 14 (9.3%), 10 (8.5%), *P*=0.945]; and body mass index (28.65±4.18, 28.58±4.30, 28.41±4.21, *P*=0.794) (Table 1).

**Table 1:** Baseline characteristics of patients. Femoral vs. left radial and right radial vs. left radial group.

	Femoral (n=174)	Left Radial (n=148)	*P*	Left Radial (n=148)	Right Radial (n=120)	*P*
Female gender	45 (25.9%)	42 (28.4%)	0.612*	42 (28.4%)	22 (18.3%)	0.062*
Age	67.03±7.95	68.55±9.72	0.123^#^	68.55±9.72	67.13±9.49	0.231^#^
Hypertension	156 (89.7%)	132 (89.2%)	0.892^	132 (89.2%)	109 (90.8%)	0.689^
Diabetes mellitus	92 (52.9%)	75 (50.7%)	0.694^	75 (50.7%)	62 (51.7%)	0.903^
Hyperlipidemia	148 (81.1%)	123 (83.1%)	0.633^	123 (83.1%)	91 (75.8%)	0.168^
Renal failure	4 (2.3%)	4 (2.7%)	0.817*	4 (2.7%)	7 (5.8%)	0.228*
Smokers	16 (9.2%)	14 (9.3%)	0.948*	14 (9.3%)	10 (8.5%)	0.748*
Weight	81.89±12.78	81.10±13.10	0.583^#^	81.10±13.10	81.63±13.56	0.748^#^
Height	1.67±0.10	1.67±0.12	0.823^#^	1.67±0.12	1.67±0.11	0.684^#^
BMI	28.65±4.18	28.58±4.30	0.885^#^	28.58±4.30	28.41±4.21	0.757^#^

* They were analyzed by Fisher's Exact test,

^ Chi-Square test.

# Independent Samples T test. BMI=Body Mass Index

**Fig. 4:**
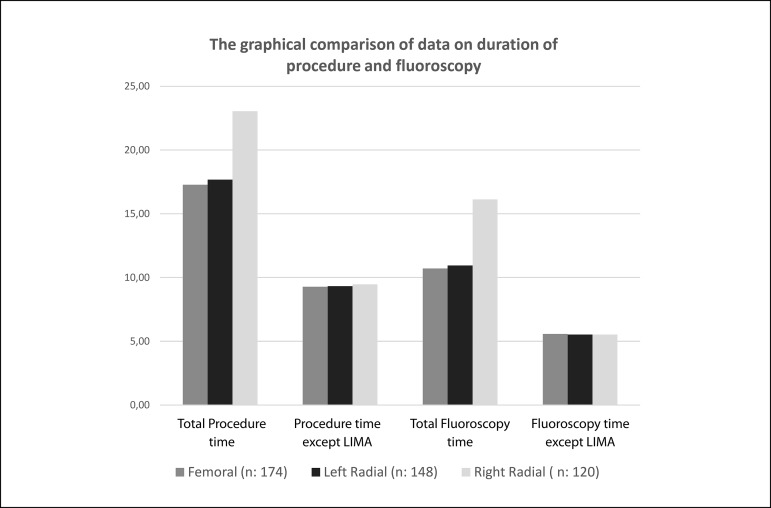
The graphical comparison of data on duration of procedure and fluoroscopy.

The femoral and left radial groups were compared in terms of primary endpoints. The femoral access was found to be significantly better in terms of total procedure time (17.28±1.68 min *vs*. 17.68±2.34 min, *P*<0.001) and fluoroscopy time (10.71±1.65 *vs*. 10.94±1.25, *P*=0.007), but similar in terms of amount of contrast media (59.00±9.31 *vs*. 58.27±11.84, *P*=0.530) (Table 2, Figure 4).

**Table 2:** Catheter based results. Femoral vs. left radial group and left radial vs. right radial group.

Variables		Femoral (n=174)	Left Radial (n=148)	Right Radial (n=120)	*P*
Total procedure time (min)		17.28±1.68	17.68±2.34	23.04±5.84	<0.001*
Total procedure time except LIMA (min)		9.28±1.38	9.32±1.03	9.47±1.01	0.304*
Fluoroscopy time (min)		10.71±1.65	10.94±1.25	16.12±5.28	0.001*
Fluoroscopy time except LIMA (min)		5.58±0.92	5.53±0.86	5.53±0.60	0.545*
Amount of contrast media used		59.00±9.31	58.27±11.84	61.04±11.88	0.102*
LIMA graft effectivity		129 (74.1%)	108 (73.1%)	91 (75.8%)	0.868
LIMA selective imaging		147 (85.5%)	116 (78.4%)	88 (73.3%)	0.456
Local hematoma		12 (6.9%)	3 (2%)	5 (4.2%)	0.306
Radial artery spasm		__	16 (10.8%)	14 (11.7%)	0.825
Radial artery occlusion^#^		__	4 (2.7%)	4 (3.3%)	0.763
Pseudoaneurysm^#^		4 (2.3%)	__	__	0.999
Allergic reaction		6 (3.4%)	5 (3.4%)	4 (3.3%)	0.984
Hypotension		12 (6.9)	10 (6.8)	9 (7.5%)	0.814
Opaque nephropathy^#^		__	1 (0.7%)	__	0.367
Major bleeding		__	__	__	__
Ao-Saphenous vein graft selective imaging	Ao-LAD or Diagonal	67 (88.15%) n=76^2^	71 (92.20%) n=77^1^	53 (86.88%) n=61^1^	0.236*
	Ao- OM or IM	85 (90.00%) n=92^4^	82 (92.13%) n=89^3^	72 (90.00%) n=80^3^	0.088*
	Ao- RCA	73 (91.25%) n=80^6^	87 (92.55%) n=94^5^	72 (91.13%) n=79^5^	0.073*

1 Ao-LAD or Diagonal (aorta to left anterior descending or diagonal artery) graft count in the right radial group.

2 Ao-LAD or Diagonal graft count in the left radial group.

3 Ao-Om or IM (aorta to obtuse marginal or intermediary artery) graft count in the right radial group.

4 Ao-Om or IM count in the left radial group.

5 Ao-RCA (aorta to right coronary artery) graft count in the right radial group.

6 Ao-RCA count in the left radial group.

# These data were recorded in the examination performed one month after the procedure.

* They were analyzed by one way ANOVA test (95% confidence interval).

The left and right radial groups were compared in terms of primary endpoints. Left radial access was found to be significantly better in terms of total procedure time (17.68±2.34 min *vs*. 23.04±5.84 min, *P*=0.001) and fluoroscopy time (10.94±1.25 *vs*. 16.12±5.28 min, *P*<0.038), but similar in terms of the amount of contrast media (58.27±11.84 *vs*. 61.04±11.88 mlt, *P*=0.530) (Table 2 and Figure 4).

Total procedure time, with the exception of LIMA imaging, was similar in the right radial left radial, and femoral, groups, respectively (9.47±1.03, 9.32±1.01, 9.28±1.38 min, *P*=0.304). Similarly, total fluoroscopy time, with the exception of LIMA imaging, was comparable in all three groups (5.58±0.92, 5.53±0.86, 5.53±0.60 min, *P*=0.641) (Table 2, Figure 4).

Graft imaging success was compared between the femoral and left radial group. Even if LIMA is not used as a graft, LIMA is visualized in each case. LIMA selective imaging proportion (85.5% *vs*. 78.4%, *P*=0.813), the success of selective imaging of aorto-left anterior descending artery (Ao-LAD) or diagonal (88.15% n=76 *vs*. 92.20% n=77 *P*=0.229), aorta-obtuse marginal artery (Ao-OM) or intermediary artery (IM) (90.00% n=92 *vs*. 92.13% n=89, *P*=0.424), aorta-right coronary artery (Ao-RCA) (91.25% n=80 *vs*. 92.55% n=94 *P*=0.423) were found to be similar in three groups (Table 2 and Figure 4).

Graft imaging success was compared between left radial and right radial group. LIMA selective imaging proportion [116 (78.4%, n=148) *vs*. 88 (73.3%, n=120), *P*=0.456]; success of selective imaging of Ao-LAD or diagonal [71 (92.20%, n=77) *vs*. 53 (86.88%, n=61) *P*=0.579]; Ao-OM or IM [82 (92.13%, n=89); *vs*. 72(90.00% n=80, *P*=0.484]; Ao-RCA [87 (92.55% n=94); and *vs*. 72 (91.13%, n=79), *P*=0.873] were been found to be similar in both groups (Table 2).

There were significant differences in total procedure time and total fluoroscopy time among the three groups from the results of "one-way ANOVA test". Then, these continuous variables were compared by "*post hoc* analysis" with the "Bonferroni method". According to *post hoc* analysis results, there was no significant difference between femoral access and left radial, right radial access and left radial access groups whereas, a significant difference was found between the right radial access and femoral access in terms of these two continuous variables (Figures 3 and 4).

There was no difference between the femoral and left radial groups in terms of major bleeding, pseudoaneurysm, allergic reaction, hypotension, opaque nephropathy, and major bleeding (Table 2).

There were no significant differences between the left radial and right radial access groups in terms of radial artery spasm, radial artery occlusion, pseudoaneurysm, allergic reaction, hypotension, opaque nephropathy and major bleeding (Table 2).

## DISCUSSION

With the spread of angiography and post-CABG surgery operations and the increase in medical developments and the number of experienced physicians, the number of post-CABG surgery patients in the community is gradually increasing.

Because of the increasing number of post-CABG surgery patients and the widespread use of the transradial access in angiogaphy procedure, the preference rate of the radial access in the post-CABG surgery angiography has also increased. For example, in our clinic, five years ago, one in 22 cases of angiography was a post-CABG surgery patient.

Coronary angiography and the percutaneous vascular interventions are performed to compare the right radial access versus the left radial access. In our opinion, the transradial coronary and peripheral angiographies are still in development.

In the first few years, bypass angiographies performed by the femoral route started to be performed with the spread of the radial pathway. In the first studies of comparing the left radial with the femoral route, the duration of the left radial route and complication development were similar^[20]^.

In the post-CABG angiography procedure, the first preferred radial route is the radial access, since it resembles the femoral route. However, most of the coronary angiographies are performed on the right radial route due to the frequent occurrence of radial artery occlusion, the use of the left radial artery as a graft, because the angiography table is usually designed from the right side, and the angiography laboratories are better suited for right-handed operators^[21,22]^.

Therefore, the right radial access experience and preference can be said to be higher than the left. The results of this study, in which we investigated the feasibility of post-CABG angiography performed using the right radial access, suggests that the right radial pathway may be preferred when imaging LIMA grafts when compared with the left radial and femoral access. Except for LIMA graft imaging, no statistically significant difference was found between the right radial access with femoral and left radial access in terms of the graft imaging success, the procedure time and complication development.

In our web-based research, we found a non-comparative series of two cases and a case report to show that a post-CABG could be displayed safely with the right radial route^[23-25]^. Coronary angiography with right radial access is the most preferred way by operators today. In order to contribute to the development of the post-CABG angiography method by right radial route, we compare our experiences of the right radial access with the left radial access and the left radial access with the femoral access in the bypass grafts imaging.

According to the results obtained, the femoral path was better in terms of total procedure time and fluoroscopy time. However, it can be estimated that these times will improve in the future with increased experience.

In our study, femoral access and the left radial access were better than the right radial access, but this difference was due to LIMA imaging. The amount of opaque substance used is similar to the femoral route. This difference may be attributed to the use of a 6F catheter on the femoral route.

Another purpose of this study was to show that it is possible and safe to perform the post-CABG angiography with right radial route. For this purpose, the left radial access was compared with the right radial access, and the time of procedure with the left radial, fluoroscopy time was better than the right radial path with a slight time difference.

## CONCLUSION

There is no difference in the post-CABG angiography in the left radial and right radial routes, except in terms of LIMA imaging. When there is no LIMA graft, the graft angiography performed with the left radial route *versus* the right are similar. Patients with LIMA can also undergo post-CABG angiography on the right transradial route; duration of the procedure may be longer than the left, but there is no difference with respect to left radial route in terms of complication development and patient comfort.

Radial artery occlusion, hematoma, radial artery spasm, left radial and right radial access are not different. Nevertheless, the femoral path is disadvantageous according to radial pathway in terms of comfort loss, hematoma, and other complications.

**Table:** 

Authors' roles & responsibilities	
YB	Conception or design of the work; acquisition, analysis, or interpretation of data for the work; drafting the work or revising it critically for important intellectual content; final approval of the version to be published
MHA	Conception or design of the work; acquisition, analysis, or interpretation of data for the work; drafting the work or revising it critically for important intellectual content; final approval of the version to be published
MLA	Conception or design of the work; acquisition, analysis, or interpretation of data for the work; drafting the work or revising it critically for important intellectual content; final approval of the version to be published
AÖ	Conception or design of the work; acquisition, analysis, or interpretation of data for the work; drafting the work or revising it critically for important intellectual content; final approval of the version to be published
